# Pharmacovigilance study of the frequency of gastrointestinal ulceration reports associated with immune checkpoint inhibitors: insights from the FDA adverse event reporting system

**DOI:** 10.3389/fphar.2025.1682259

**Published:** 2025-11-12

**Authors:** Liqiang Lei, Lifen Wang, Sugang Shen, Ge Zhao, Xingming Zhao, Honglin Dong

**Affiliations:** 1 Department of General Surgery, The Second Hospital of Shanxi Medical University, Taiyuan, Shanxi, China; 2 Department of Respiratory Medicine, The Fourth People’s Hospital of Taiyuan, Taiyuan, Shanxi, China; 3 Department of Urology, The Second Hospital of Shanxi Medical University, Taiyuan, Shanxi, China; 4 Department of Vascular Surgery, The Second Hospital of Shanxi Medical University, Taiyuan, Shanxi, China

**Keywords:** gastrointestinal ulceration, immune checkpoint inhibitor, adverse events, safety, FAERS

## Abstract

**Background:**

Immune checkpoint inhibitors (ICIs) have revolutionized the treatment landscape for cancer, yet they are linked to immune-related adverse events (AEs), one of which includes gastrointestinal ulceration (GU). The objective of this study was to evaluate the reporting frequency of GU reported in connection with ICIs by utilizing data from the United States (U.S.) Food and Drug Administration Adverse Event Reporting System (FAERS).

**Materials and methods:**

AEs pertaining to GU attributed to ICIs were extracted from the U.S. FAERS database for the time frame spanning from the fourth quarter of 2018 to the fourth quarter of 2024. A disproportionality analysis was performed employing the reporting odds ratio (ROR) and information component (IC), accompanied by 95% confidence intervals (95% CI). Beyond the disproportionality analysis, this investigation also examined gender disparities and the latency period for the onset of gastrointestinal AEs related to ICIs.

**Results:**

A total of 1,415 adverse event reports regarding GU linked to ICIs as the primary suspect drug were collected. The occurrence of GU attributed to ICIs was observed to be more prevalent among males and older individuals. The principal countries reporting these events were Japan, the United States, and Germany, with Japan notably contributing the highest number of reports for Nivolumab and Pembrolizumab. A significant association was observed between the drugs Ipilimumab (ROR: 3.73 [3.14–4.44], IC: 1.89), Pembrolizumab (ROR: 3.08 [2.83–3.35], IC: 1.60), Atezolizumab (ROR: 4.10 [3.67–4.58], IC: 2.02), Nivolumab (ROR: 2.45 [2.23–2.69], IC: 1.28) and reports of GU. Conversely, Cemiplimab (ROR: 1.06 [0.55–2.05], IC: 0.09) did not exhibit a significant correlation. Among patients administered Pembrolizumab, the reporting frequency of intestinal perforation in females was considerably greater than that in males, presenting the most robust signal strength (ROR = 1.98 [1.28–3.05], P < 0.01).

**Conclusion:**

This study presents the most current real-world evidence regarding the safety profile of ICIs therapy in relation to GU, highlighting variations among different ICIs and between genders. It is imperative for healthcare providers to maintain heightened awareness of potential GU adverse events associated with ICIs and to implement timely preventive or therapeutic interventions to enhance the safety of ICIs in clinical practice.

## 1 Introduction

Immune checkpoint inhibitors (ICIs) are a new class of drugs that enhance the immune system’s ability to fight tumors by blocking immune checkpoint proteins, such as programmed cell death protein 1 (PD-1) and its ligand (PD-L1), as well as cytotoxic T lymphocyte-associated protein 4 (CTLA-4). These targets are typically inhibited using monoclonal antibodies, which have markedly improved outcomes in various advanced cancers, including gastrointestinal, lung, and renal cancers, and melanoma, with their use becoming increasingly integrated into clinical practice (Terrin et al., 2023). However, the use of ICIs often comes with immune-related adverse events (AEs), with gastrointestinal AEs being among the most common and potentially serious complications (Alomari et al., 2022; Shatila et al., 2024).

In recent years, the use of ICIs like Pembrolizumab and Nivolumab has increased significantly, leading to a heightened awareness of gastrointestinal ulceration (GU) associated with these therapies. While lower gastrointestinal issues, such as diarrhea and colitis, have been well-documented (Sugiyama et al., 2022; Shrivastava and Patel, 2024), upper gastrointestinal AEs, including gastritis, gastric ulcers, and duodenal ulcers are less common and occur at a lower incidence rate. The clinical features and management strategies for these complications are not well understood (Hayama et al., 2020; Ferrian et al., 2021; Panneerselvam et al., 2021). For example, gastritis and gastric ulcers related to Pembrolizumab are rare but can present with symptoms like upper abdominal discomfort, gastroesophageal reflux, and signs typical of peptic ulcers. Standard treatments, such as proton pump inhibitors, often fall short in these cases, leading to the need for more aggressive interventions like systemic corticosteroids or biological therapies, including infliximab, for effective management (Liu et al., 2022). Similarly, Nivolumab therapy may lead to duodenal ulcers, which can be detected as mucosal damage during endoscopic examinations (Reddy et al., 2023). The underlying cause of these ulcerative lesions is thought to be linked to immune-mediated inflammatory responses resulting from tissue damage caused by T cell activation through ICIs (Au et al., 2024; Gupta et al., 2024). However, the understanding of the pathophysiology, risk factors, and long-term outcomes associated with these conditions is still quite limited (Haryal et al., 2023). Despite the relatively low occurrence of ICIs-induced GU, the increasing rates of various cancers and the growing use of ICIs highlight a significant concern. Patients who respond well to ICIs may enjoy prolonged survival, which raises the likelihood of encountering ICIs-related GU, emphasizing the importance of their recognition and management.

The Food and Drug Administration Adverse Event Reporting System (FAERS) database comprises individual case safety reports submitted by both healthcare professionals and non-healthcare entities, as well as pharmaceutical companies. Analyses derived from FAERS provide valuable real-world evidence to explore clinical characteristics and risk factors of drug-associated adverse events. The objective of this study was to evaluate the reporting frequency of GU reported in connection with ICIs by utilizing data from FAERS.

## 2 Materials and methods

### 2.1 Data sources and collection

Data for this investigation were extracted utilizing an interactive and publicly accessible online platform known as the FAERS Public Dashboard (https://www.fda.gov/drugs/questions-and-answers-fdas-adverse-event-reporting-system-faers/fda-adverse-event-reporting-system-faers-public-dashboard). This study encompasses all recorded AEs associated with ICIs from the fourth quarter of 2018 through the fourth quarter of 2023. Each quarterly dataset consists of seven distinct sub-files, which include demographic data (DEMO), drug-related information (DRUG), adverse events (AEs) (REAC), patient outcomes (OUTC), sources of the reports (RPSR), therapy dates (THER), and indications (INDI).

**FIGURE 1:**
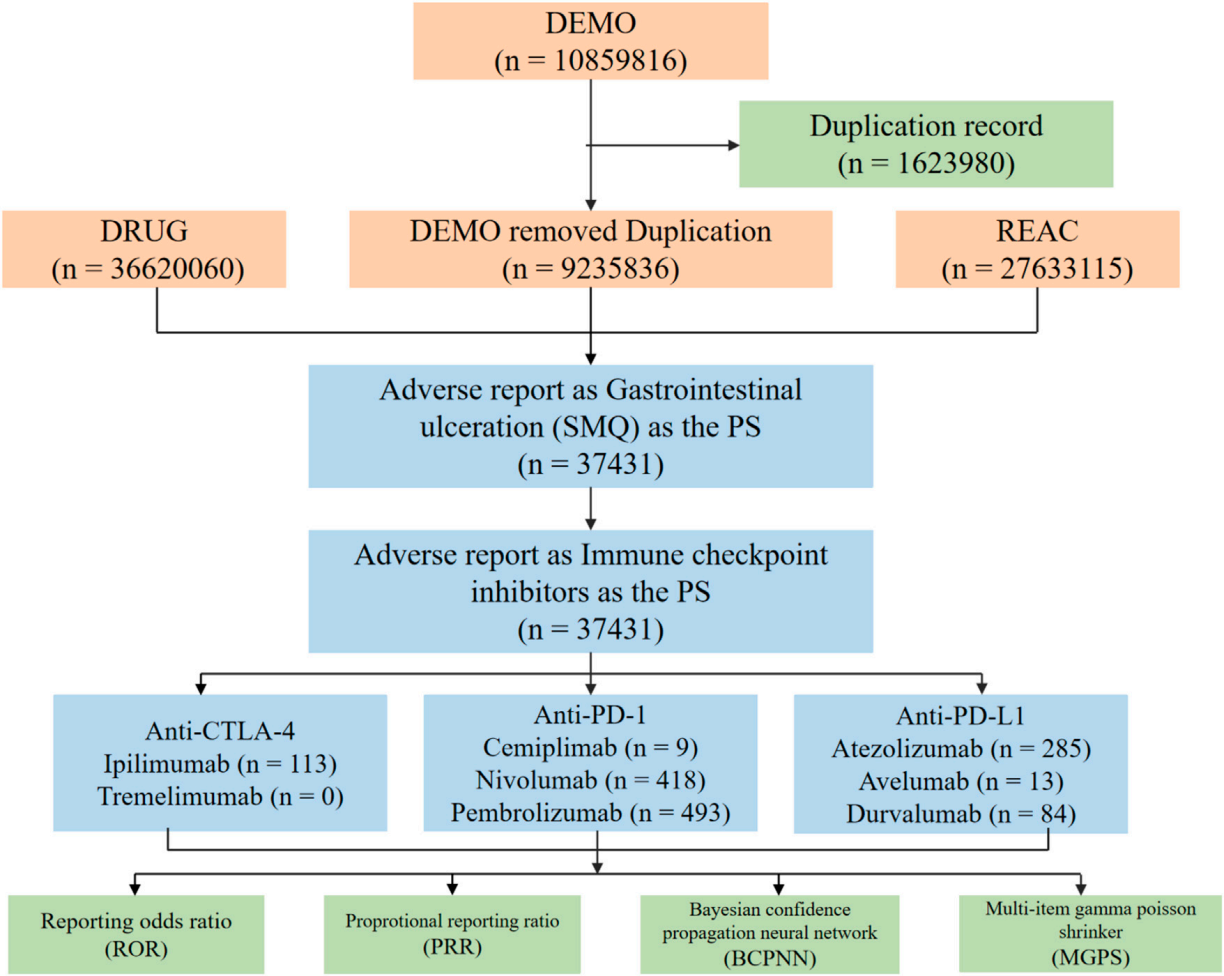
The FAERS database’s pipeline flowchart for screening immune checkpoint inhibitors-associated gastrointestinal ulceration.

The current analysis employed both brand and generic names of the medications to identify relevant reports concerning ICIs. All AEs were categorized according to the Medical Dictionary for Regulatory Activities (MedDRA 27.1). Given the extensive number of preferred terms (approximately 25,000) and their restricted specificity, standardized MedDRA queries (SMQs) were formulated. SMQs consist of standardized MedDRA terms that pertain to specific medical conditions, thereby enhancing data retrieval and signal identification. The final study cohort was defined by applying specific inclusion criteria. We included unique case reports that met both of the following conditions: (1) contained one or more Preferred Terms from the “Gastrointestinal Ulceration” SMQ, and (2) listed an immune checkpoint inhibitor as the “Primary Suspect” drug. To ensure data accuracy and mitigate the risk of duplicate entries, a data cleansing process was performed prior to the analysis, as detailed in Figure 1. Initially, duplicates were eliminated in accordance with FDA guidelines, preserving only the most recent version of each case or report, which is defined by the latest submission date, to maintain data uniqueness. Furthermore, only those adverse event cases where the drug role was designated as “Primary Suspect” were included, thereby excluding entries with “concomitant” or “interaction” roles. Since this study utilized publicly accessible data, the entire research process did not necessitate approval from an institutional review board or informed consent from patients, given that the data were anonymized and publicly available.

### 2.2 Data analysis

Disproportionality analysis techniques serve as essential analytical instruments frequently utilized in the domain of pharmacovigilance; however, a standardized methodology for the identification of safety signals has yet to be established. In order to enhance the reliability of the assessment concerning the relationship between pharmaceuticals and AEs, this investigation implemented two disproportionality analysis techniques: the reporting odds ratio (ROR) and the Bayesian confidence propagation neural network (BCPNN), alongside their respective 95% confidence intervals (CIs). These methodologies were employed to measure the association between ICIs and AEs associated with gastrointestinal ulceration (GU). The formulas for these analytical methods and the criteria for signal detection are detailed in Table 1. Specifically, a result is deemed statistically significant when the lower limit of the 95% CI for ROR exceeds 1, provided that the report count is no fewer than three; similarly, a result is considered statistically significant when the lower limit of the information component’s 95% CI (IC025) surpasses 0. If both disproportionality analysis methods meet these specified conditions, then a notable safety signal for a specific adverse event can be inferred. Typically, an elevated disproportionality measure value correlates with a more pronounced signal, indicating a stronger relationship between the drug and the occurrence of AEs.

**TABLE 1:** Two major algorithms used for signal detection.

Algorithms	Equation	Criteria
ROR	$\text{ROR}=\frac{a/c}{b/d}$	Lower limit of 95% CI > 1, N ≥ 3
	$95\%\text{CI}=e^{\text{ln}\left(ROR\right)\pm 1.96\sqrt{\frac{1}{a}+\frac{1}{b}+\frac{1}{c}+\frac{1}{d}}}$	
BCPNN	$\text{IC}={\text{log}}_{2}\frac{a\left(a+b+c+d\right)}{\left(a+b\right)\left(a+c\right)}$	*IC*025 > 0
	$\text{IC}025=E\left(IC\right)-2\sqrt{V\left(IC\right)}$	

Abbreviations: ROR, reporting odds ratio; BCPNN, bayesian confidence propagation neural network; IC, information component; IC025, the lower limit of 95% CI, of the IC; E (IC), the IC, expectations; V(IC), the variance of IC; a, number of reports containing both the target drug and target adverse drug reaction; b, number of reports containing other adverse drug reaction of the target drug; c, number of reports containing the target adverse drug reaction of other drugs; d, number of reports containing other drugs and other adverse drug reactions. 95%CI, 95% confidence interval; N, the number of reports.

### 2.3 Secondary analysis

To investigate the influence of gender on AEs associated with ICIs, this research compared the reports of GU-related AEs among male and female patients following ICIs treatment. Furthermore, to evaluate the risk of gender-specific adverse drug reactions, ROR signal analysis was employed to analyze the disproportionality analysis outcomes between male and female patients after ICIs administration. Specifically, when the lower limit of ROR is greater than 1 and the P value derived from the chi-square test is less than 0.05, this indicates an increased risk of AEs in males. Conversely, if the upper limit of ROR is below 1 and the P value from the chi-square test is less than 0.05, this suggests a heightened risk of AEs in females. To control for the increased risk of Type I errors due to multiple comparisons, a Bonferroni correction was applied to the disproportionality analysis.

### 2.4 Statistical analysis

All statistical evaluations and data mining procedures were performed using R software (version 4.4.1) and Microsoft Excel 2019. The chi-square test was utilized to compare the occurrence of GU AEs related to ICIs between male and female patients. Additionally, this study adhered strictly to the READUS-PV guidelines designed specifically to standardize signal detection within the FAERS database (Bate et al., 1998).

## 3 Results

### 3.1 Descriptive analysis

An analysis was conducted on a total of 10,859,816 records from the DEMO database, covering the period from the fourth quarter of 2018 through to the fourth quarter of 2024. After eliminating 1,623,980 duplicate entries, 9,235,836 records remained for further study. By integrating data from the DRUG and REAC databases, 37,431 adverse event reports were isolated, utilizing GU (Standardized MedDRA Query, SMQ) as the preferred term (PT). The subsequent analysis concentrated on reports associated with ICIs. Among these reports, drugs classified under the Anti-CTLA-4 category included Ipilimumab (n = 113) and Tremelimumab (n = 0). The Anti-PD-1 category featured Cemiplimab (n = 9), Nivolumab (n = 418), and Pembrolizumab (n = 493). In the Anti-PD-L1 classification, Atezolizumab (n = 285), Avelumab (n = 13), and Durvalumab (n = 84) were recorded.

The distribution of immune checkpoint inhibitors (ICIs) varied by gender, age, reporter type, outcome, and reporting country (Table 2). A greater number of male patients reported AEs compared to female patients for the majority of drugs: for instance, Atezolizumab (52.6%), Avelumab (76.9%), and Cemiplimab (55.6%) exemplify this trend. In terms of age distribution, individuals aged between 65 and 85 years represented a more significant proportion of reports for most drugs, with Ipilimumab reaching 50.4%. Notably, Nivolumab recorded 41 reports within the <18 age category, which was higher than that of the other medications. The majority of reports were submitted by healthcare professionals, particularly for Atezolizumab (82.5%), Avelumab (76.9%), and Cemiplimab (66.7%). Regarding outcomes, fatalities were documented for all drugs, with Ipilimumab exhibiting the highest mortality rate at 30.1%. The majority of the reports originated from Japan, the United States, and Germany, with a notably higher frequency of reports for Nivolumab and Pembrolizumab coming from Japan.

**TABLE 2:** Characteristics of gastrointestinal ulceration associated with Immune Checkpoint Inhibitors (ICIs).

Total	Available cases, N (%)						
	Atezolizumab (N = 285)	Avelumab (N = 13)	Cemiplimab (N = 9)	Durvalumab (N = 84)	Nivolumab (N = 418)	Pembrolizumab (N = 493)	Ipilimumab (N = 113)
Gender							
F	79 (27.7%)	3 (23.1%)	1 (11.1%)	20 (23.8%)	118 (28.2%)	270 (54.8%)	30 (26.5%)
M	150 (52.6%)	10 (76.9%)	5 (55.6%)	48 (57.1%)	269 (64.4%)	216 (43.8%)	66 (58.4%)
Unknown	56 (19.6%)	0 (0%)	3 (33.3%)	16 (19.0%)	31 (7.4%)	7 (1.4%)	17 (15.0%)
Age							
<18	1 (0.4%)	0 (0%)	0 (0%)	2 (2.4%)	6 (1.4%)	41 (8.3%)	0 (0%)
>85	0 (0%)	2 (15.4%)	0 (0%)	0 (0%)	2 (0.5%)	5 (1.0%)	1 (0.9%)
18–64.9	82 (28.8%)	4 (30.8%)	1 (11.1%)	20 (23.8%)	153 (36.6%)	143 (29.0%)	25 (22.1%)
65–85	122 (42.8%)	5 (38.5%)	3 (33.3%)	37 (44.0%)	178 (42.6%)	168 (34.1%)	57 (50.4%)
Unknown	80 (28.1%)	2 (15.4%)	5 (55.6%)	25 (29.8%)	79 (18.9%)	136 (27.6%)	30 (26.5%)
Reporters							
Consumer	5 (1.8%)	0 (0%)	0 (0%)	4 (4.8%)	35 (8.4%)	79 (16.0%)	2 (1.8%)
Health professional	36 (12.6%)	2 (15.4%)	3 (33.3%)	5 (6.0%)	108 (25.8%)	32 (6.5%)	32 (28.3%)
Physician	235 (82.5%)	10 (76.9%)	6 (66.7%)	64 (76.2%)	205 (49.0%)	353 (71.6%)	63 (55.8%)
Other health-professional	0 (0%)	0 (0%)	0 (0%)	1 (1.2%)	47 (11.2%)	4 (0.8%)	7 (6.2%)
Pharmacist	7 (2.5%)	1 (7.7%)	0 (0%)	3 (3.6%)	23 (5.5%)	25 (5.1%)	9 (8.0%)
Unknown	2 (0.7%)	0 (0%)	0 (0%)	7 (8.3%)	0 (0%)	0 (0%)	0 (0%)
Outcomes							
Death	82 (28.8%)	2 (15.4%)	3 (33.3%)	24 (28.6%)	128 (30.6%)	125 (25.4%)	34 (30.1%)
Disability	1 (0.4%)	0 (0%)	0 (0%)	0 (0%)	2 (0.5%)	0 (0%)	0 (0%)
Hospitalization-initial or prolonged	99 (34.7%)	5 (38.5%)	4 (44.4%)	21 (25.0%)	145 (34.7%)	175 (35.5%)	30 (26.5%)
Life-threatening	22 (7.7%)	0 (0%)	0 (0%)	24 (28.6%)	55 (13.2%)	35 (7.1%)	15 (13.3%)
Other serious (important medical events)	61 (21.4%)	6 (46.2%)	2 (22.2%)	10 (11.9%)	76 (18.2%)	132 (26.8%)	31 (27.4%)
Unknown	20 (7.0%)	0 (0%)	0 (0%)	5 (6.0%)	12 (2.9%)	26 (5.3%)	3 (2.7%)
Reporter countries							
United states	53 (18.6%)	3 (23.1%)	1 (11.1%)	5 (6.0%)	74 (17.7%)	46 (9.3%)	25 (22.1%)
Japan	98 (34.4%)	4 (30.8%)	1 (11.1%)	43 (51.2%)	162 (38.8%)	305 (61.9%)	60 (53.1%)
Germany	19 (6.7%)	2 (15.4%)	1 (11.1%)	4 (4.8%)	36 (8.6%)	19 (3.9%)	2 (1.8%)
China	18 (6.3%)	0 (0%)	0 (0%)	3 (3.5%)	14 (3.3%)	8 (1.6%)	0 (0%)
Other countries	97 (34.0%)	4 (30.8%)	6 (66.7%)	29 (34.5%)	132 (31.6%)	115 (23.3%)	26 (23.0%)

### 3.2 Disproportionality analysis

Table 3 presents the analysis of GU reporting signals across various ICIs. The findings revealed that Atezolizumab exhibited a reporting odds ratio (ROR) of 4.10 (95% confidence interval [CI]: 3.67–4.58) and an information component (IC) of 2.02 (IC025 = 1.86). Avelumab presented an ROR of 2.42 (95% CI: 1.46–4.02) and an IC of 1.27 (IC025 = 0.55). For Durvalumab, the ROR was calculated at 2.55 (95% CI: 2.08–3.13), accompanied by an IC of 1.35 (IC025 = 1.05). Cemiplimab, however, reported an ROR of 1.06 (95% CI: 0.55–2.05) with an IC of 0.09 (IC025 = −0.82). Nivolumab’s ROR was 2.45 (95% CI: 2.23–2.69) and its IC was 1.28 (IC025 = 1.15). Pembrolizumab exhibited an ROR of 3.08 (95% CI: 2.83–3.35) and an IC of 1.60 (IC025 = 1.48). Lastly, Ipilimumab exhibited an ROR of 3.73 (95% CI: 3.14–4.44) and an IC of 1.89 (IC025 = 1.64). Based on the criteria established for disproportionality analysis—specifically, an ROR lower confidence interval exceeding 1 and a positive IC025—these findings suggest that Ipilimumab, Pembrolizumab, Atezolizumab, and Nivolumab are significantly correlated with an increased risk of GU, whereas Cemiplimab did not demonstrate a statistically significant association.

**TABLE 3:** Signal detection for ICI-associated gastrointestinal ulceration.

Drug	N	ROR	ROR 95%CI	IC	IC025
Atezolizumab	316	4.1	3.67–4.58	2.02	1.86
Avelumab	15	2.42	1.46–4.02	1.27	0.55
Durvalumab	93	2.55	2.08–3.13	1.35	1.05
Cemiplimab	9	1.06	0.55–2.05	0.09	−0.82
Nivolumab	459	2.45	2.23–2.69	1.28	1.15
Pembrolizumab	553	3.08	2.83–3.35	1.6	1.48
Ipilimumab	131	3.73	3.14–4.44	1.89	1.64

N, number; ROR, reporting odds ratio; 95%CI, 95% confidence interval; IC, information component; IC025, the lower limit of 95% CI, of the IC.

Supplementary Table S1 provides a detailed breakdown of the specific AEs and associated statistical metrics for each drug. Notably, Atezolizumab displayed markedly elevated ROR values for several AEs, including ileal perforation (ROR = 15.55, 95% CI: 6.91–35.02), jejunal perforation (ROR = 16.50, 95% CI: 5.23–52.06), and small bowel fistula (ROR = 7.28, 95% CI: 2.33–22.76). Similarly, Ipilimumab documented high ROR values for AEs such as colonic perforation (ROR = 12.76, 95% CI: 8.79–18.52), gastrointestinal perforation (ROR = 10.61, 95% CI: 6.27–17.96), and peritonitis (ROR = 4.09, 95% CI: 2.61–6.42). Noteworthy ROR values were also observed for Nivolumab and Pembrolizumab across specific adverse event categories. Nivolumab reported an ROR of 5.48 (95% CI: 3.43–8.74) for jejunal perforation and 5.15 (95% CI: 3.99–6.65) for colonic perforation, while Pembrolizumab revealed an ROR of 16.89 (95% CI: 12.71–22.45) for jejunal perforation and 5.26 (95% CI: 4.06–6.81) for colonic perforation.

### 3.3 Gender differences in AEs

**FIGURE 2:**
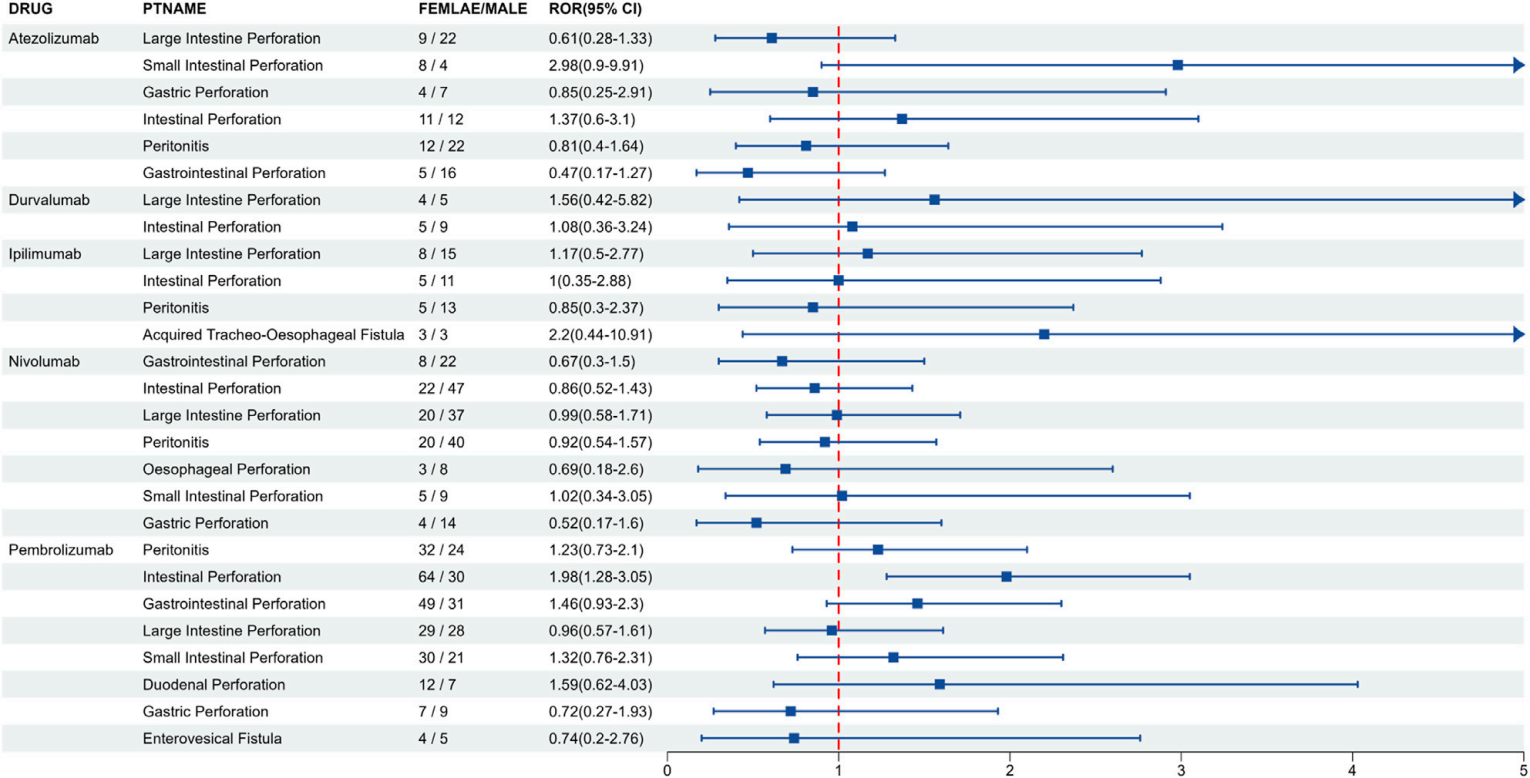
Gender-based risk signal analysis of adverse events related to gastrointestinal ulceration with immune checkpoint inhibitors.

To investigate the gender-specific variations in gastrointestinal AEs associated with ICIs, the Reporting Odds Ratio (ROR) algorithm was employed to assess the signal strength of various gastrointestinal-related AEs across male and female populations. The analysis recorded 40 ICIs-related AEs were recorded in females (as detailed in Supplementary Table S2), whereas a higher count of 54 ICIs-related AEs was noted in males (refer to Supplementary Table S3). A total of twenty-seven AEs were common to all genders, which included instances of gastric and intestinal perforations. Notably, several AEs were exclusive to females, such as rectal perforation (n = 12, ROR = 42.70 [23.15–78.79]), pneumoperitoneum (n = 7, ROR = 5.95 [2.81–12.59]), duodenal perforation (n = 12, ROR = 19.67 [10.93–35.40]), and diverticular perforation related to Nivolumab (n = 6, ROR = 7.12 [3.18–15.98]). Conversely, specific AEs, including spontaneous bacterial peritonitis (n = 5, ROR = 31.20 [12.52–77.75]), retroperitoneal abscess (n = 3, ROR = 18.87 [5.71–62.35]), and colonic fistula (n = 3, ROR = 4.20 [1.33–13.30]), were exclusively observed in males. Furthermore, in the cohort treated with Pembrolizumab, the occurrence of intestinal perforation was notably more prevalent in females, being more than double that of the male cohort (females: n = 64; males: n = 30), with a pronounced signal strength (ROR = 1.98 [1.28–3.05], P < 0.01) (see Supplementary Table S4; Figure 2).

### 3.4 Temporal distribution of AEs

The frequency of AEs associated with seven different ICIs exhibited variability over distinct temporal intervals. During the initial treatment phase (days 0–30), a high proportion of reports was observed for most drugs: Atezolizumab at 42.86%, Nivolumab at 40.00%, Pembrolizumab at 35.00%, Avelumab at 46.15%, Cemiplimab at 50.00%, Ipilimumab at 44.44%, and Durvalumab at 42.86%. As the treatment duration progressed, the frequency of reported AEs demonstrated a gradual decline, albeit with notable fluctuations during certain intervals. For instance, Ipilimumab exhibited an adverse event reporting rate of 22.22% between days 31–60, which further reduced to 11.11% during days 91–120, while Durvalumab revealed a reporting rate of 9.52% in the days 151–180 timeframe. In addition, both Atezolizumab (9.52%) and Nivolumab (4.00%) continued to report AEs over an extended duration (>360 days) (as depicted in Supplementary Figure S1).

### 3.5 Sensitivity analysis

To address the potential confounding effect of medications known to increase the risk of gastrointestinal ulceration, we performed a sensitivity analysis. We excluded all case reports that explicitly listed non-steroidal anti-inflammatory drugs (NSAIDs), corticosteroids, or anticoagulants as concomitant medications. After this exclusion, the disproportionality analysis was repeated on the refined dataset (see Supplementary Tables S5, S6).

## 4 Discussion

ICIs have shown significant effectiveness in cancer treatment, but the immune-related AEs that can arise from their use are receiving increasing scrutiny. Although there is a lack of extensive research specifically focusing on gastrointestinal ulceration (GU) AEs related to ICIs, this study marks a groundbreaking real-world pharmacovigilance investigation that utilizes data from the FAERS database.

Various ICIs have different probabilities of causing GU as adverse effects. Notably, Ipilimumab, Pembrolizumab, Atezolizumab, and Nivolumab show a significantly higher likelihood of inducing such ulcers compared to other treatment options. In contrast, the association with Cemiplimab appears weak, likely due to limited clinical usage data, highlighting the need for further research to confirm its safety profile. Immune-related AEs occur as a result of an overactive immune response, which alters T cell function and cytokine production (Som et al., 2019). Normally, the PD-1/PD-L1 signaling pathway plays a vital role in regulating the immune response in mucosal tissues by preventing T cell overactivity towards commensal bacteria and dietary antigens, thereby promoting mucosal immune tolerance (Freeman et al., 2000; Pinchuk et al., 2008). However, when this pathway is blocked by medication, T cell activity is restored and significantly increased, disrupting the established immune tolerance (Balducci et al., 2021). This restoration and enhancement of T cell function lead to the infiltration of CD4^+^ and CD8^+^ T cells into gastrointestinal mucosal tissues, resulting in damage to healthy cells and the onset of inflammatory responses. Activated T cells are crucial in this inflammatory process, releasing large amounts of pro-inflammatory cytokines, such as interleukin-6 (IL-6) and tumor necrosis factor-alpha (TNF-α) (Yang et al., 2020). These cytokines play a crucial role in activating more immune cells and amplifying the inflammatory response, which can lead to damage in the gastrointestinal mucosal tissues. For instance, TNF-α can trigger apoptosis and cause harm to intestinal epithelial cells by activating the Caspase-3 apoptotic pathway (Alfen et al., 2018). When there is significant apoptosis of these epithelial cells, the structural integrity of the gastrointestinal mucosa is compromised, which reduces the effectiveness of the mucosal barrier and increases the tissue’s sensitivity to external stimuli. Additionally, the activation of the immune system may lead to changes in the intestinal immune microenvironment, further disturbing the balance of gut microbiota. This imbalance, known as dysbiosis, can worsen the damage to the gastrointestinal mucosal barrier, making it easier for inflammatory responses to develop (Raschi et al., 2020). CTLA-4 acts as a co-inhibitory receptor that is essential for preventing excessive activation of T cells by interacting with B7 molecules, thereby maintaining immune tolerance (McCoy and Le Gros, 1999). When anti-CTLA-4 monoclonal antibodies are administered, this interaction is blocked, removing the inhibitory signal and leading to increased T cell activation and proliferation. As a result, these hyperactivated T cells begin to target the gastrointestinal mucosal tissues, causing inflammatory responses and ulcerations (Buchbinder and Desai, 2016). Research has shown that after CTLA-4 blockade, there is a significant rise in the number of CD4^+^ T cells that migrate to the intestinal area. These T cells gather in the intestinal mucosa, which further exacerbates the inflammatory responses in the gut (Zhang et al., 2020). The gut microbiota plays a crucial role in influencing both the therapeutic effects and side effects associated with CTLA-4 blockade. For instance, certain gut microbial species like *Bacteroides fragilis* and Burkholderia cepacia have been found to help reduce intestinal inflammation caused by CTLA-4 inhibition (Miller and Carson, 2020). On the other hand, an imbalance or dysregulation of gut microbiota can worsen intestinal inflammation and lead to increased adverse effects following CTLA-4 blockade.

Despite a significantly higher reporting frequency of these AEs among male participants compared to females, this disparity cannot be interpreted as evidence of inherent male susceptibility to these adverse effects. The differences between genders are complex and can vary depending on the specific medication and type of adverse event. For instance, we found that the occurrence of intestinal perforation in females after receiving the PD-1 inhibitor Pembrolizumab was notably higher than in males. This suggests that females may be more sensitive to disruptions in mucosal immune homeostasis following Pembrolizumab treatment. In contrast, males reported a greater reporting frequency of gastrointestinal AEs when treated with the PD-1 inhibitor Nivolumab. The underlying biological mechanisms for these observed gender disparities remain unclear and are likely multifactorial, potentially involving differences in immune homeostasis, pharmacokinetics, or concomitant medications. Our analysis, while identifying these important clinical signals, does not contain direct patient-level data on hormone levels or immune cell phenotypes to elucidate the specific mechanisms. Therefore, the exact role of factors such as estrogen signaling or drug structure differences remains speculative and represents an important avenue for future research.

The research highlights that different drugs exhibit distinct profiles related to specific AEs. For instance, Atezolizumab is linked to a higher reporting odds ratio (ROR) for AEs such as ileal and jejunal perforation. This drug works by blocking PD-L1, which activates the immune system; however, it can also trigger immune-mediated inflammatory responses that may lead to gastrointestinal perforation (Yu et al., 2023). Similarly, Ipilimumab poses an increased risk for intestinal perforation and peritonitis. Fecher et al. pointed out that enteritis caused by Ipilimumab can resemble inflammatory bowel disease or graft-versus-host disease, showing pathological features like mucosal erosion, a rich infiltration of T cells, cryptitis, and crypt abscesses. Such immune responses can weaken the intestinal wall’s structural integrity, thereby increasing the likelihood of perforation (Fecher et al., 2013). Therefore, clinicians must be attentive to the specific risk profiles associated with these medications while monitoring and managing AEs. Since these AEs can significantly impact patient outcomes, it is recommended to conduct a thorough evaluation of patients’ gastrointestinal function before starting treatment and to perform regular gastrointestinal endoscopic assessments throughout the treatment to facilitate the early detection and management of potential adverse effects.

This investigation has several limitations that must be acknowledged. Firstly, the FAERS database is prone to underreporting, potential duplicate reports, and several reporting biases. Notably, serious or fatal adverse events are often overrepresented (selective reporting bias), which could lead to an exaggerated perception of risk. Secondly, the information in the FAERS database may be incomplete or inaccurate. Crucially, the reported mortality rates lack context and cannot be disaggregated from the high background risk of death in patients with advanced cancer; therefore, they likely overstate the risk directly attributable to ICIs and must be interpreted with caution. The lack of data on patients’ baseline characteristics, specifically the inability to account for pre-existing comorbidities such as prior gastrointestinal disease or infections, treatment dosages, and treatment durations could undermine the validity of the study’s findings. Most importantly, as an observational analysis of spontaneous reports, this study can only identify statistical associations and cannot establish causal relationships between drugs and events. Therefore, future research should focus on conducting prospective randomized controlled trials or utilizing linked electronic health records that contain detailed clinical information to clarify the causal relationship between ICIs and GU-related AEs, while also exploring the underlying mechanisms in more detail.

## 5 Conclusion

This study identifies a significant correlation between ICIs and GU-related AEs through the analysis of adverse event reports from the FAERS database, revealing variations in the reporting frequency of these events among different drug categories. These findings provide essential insights for clinicians who administer ICIs, aiding in the assessment and management of associated risks and helping to optimize personalized treatment strategies. Future studies should further investigate the mechanisms by which ICIs lead to GU and develop effective preventive and therapeutic strategies to improve the clinical safety of ICI therapies.

## Data Availability

Publicly available datasets were analyzed in this study. This data can be found here: https://www.fda.gov.
