# Supplementary material for: Pharmacovigilance study of the frequency of gastrointestinal ulceration reports associated with immune checkpoint inhibitors: insights from the FDA adverse event reporting system
Source: Front Pharmacol. 2025 Nov 12;16:1682259. doi: 10.3389/fphar.2025.1682259 (PMC12646910; doi:10.3389/fphar.2025.1682259)
Supplement: Supplementary file 1 [file DataSheet1.pdf]

Table 1 Signal Strength of Immune Checkpoint Inhibitor–Associated Gastrointestinal ulceration Events at the Preferred Term (PT) Level

	Preferred term (PTs)	N	ROR（95%CI）	IC (IC025)
Atezolizumab	ILEAL PERFORATION	6	15.55 (6.91 - 35.02)	3.92 (2.81)
	JEJUNAL PERFORATION	3	16.5 (5.23 - 52.06)	4 (2.53)
	FISTULA OF SMALL INTESTINE	3	7.28 (2.33 - 22.76)	2.85 (1.39)
	DIVERTICULAR PERFORATION	7	5.62 (2.67 - 11.84)	2.48 (1.45)
	LARGE INTESTINE PERFORATION	38	7.86 (5.71 - 10.83)	2.96 (2.49)
	SMALL INTESTINAL PERFORATION	14	10.3 (6.07 - 17.48)	3.34 (2.59)
	GASTRIC PERFORATION	13	6.34 (3.67 - 10.95)	2.65 (1.87)
	ABDOMINAL ABSCESS	12	3.57 (2.02 - 6.3)	1.83 (1.02)
	PERITONITIS	36	3.51 (2.53 - 4.88)	1.81 (1.33)
	INTESTINAL PERFORATION	35	4.42 (3.17 - 6.17)	2.13 (1.65)
	OESOPHAGEAL FISTULA	3	14.19 (4.51 - 44.64)	3.79 (2.32)
	GASTRIC ULCER PERFORATION	3	3.43 (1.1 - 10.68)	1.77 (0.32)
	GASTROINTESTINAL PERFORATION	31	10.74 (7.53 - 15.33)	3.4 (2.88)
	DUODENAL PERFORATION	14	18.68 (10.97 - 31.84)	4.18 (3.42)
	ANAL ABSCESS	14	2.62 (1.55 - 4.43)	1.39 (0.64)
	GASTROINTESTINAL ANASTOMOTIC LEAK	5	16.74 (6.87 - 40.77)	4.02 (2.82)
	ANAL FISTULA	12	2.53 (1.43 - 4.46)	1.33 (0.53)
	APPENDICITIS PERFORATED	6	3.51 (1.57 - 7.83)	1.8 (0.71)
	ENTEROVESICAL FISTULA	5	10.7 (4.41 - 25.92)	3.39 (2.2)
	RECTAL PERFORATION	3	8.21 (2.62 - 25.67)	3.02 (1.56)
	SPONTANEOUS BACTERIAL PERITONITIS	8	25.83 (12.7 - 52.5)	4.63 (3.64)

Avelumab	DIVERTICULITIS INTESTINAL PERFORATED	3	7.42 (2.37 - 23.18)	2.87 (1.42)
	PERITONITIS	3	3.66 (1.18 - 11.37)	1.87 (0.43)
Cemiplimab	NA	NA	NA	NA
Durvalumab	LARGE INTESTINE PERFORATION	11	4.81 (2.66 - 8.69)	2.26 (1.42)
	SMALL INTESTINAL PERFORATION	4	6.19 (2.32 - 16.55)	2.62 (1.33)
	OESOPHAGEAL FISTULA	4	40.72 (15.02 - 110.4)	5.3 (3.98)
	INTESTINAL PERFORATION	16	4.29 (2.63 - 7.02)	2.1 (1.4)
	OESOPHAGEAL PERFORATION	3	9.59 (3.08 - 29.87)	3.25 (1.8)
	GASTROINTESTINAL PERFORATION	8	5.83 (2.91 - 11.68)	2.54 (1.57)
	SPONTANEOUS BACTERIAL PERITONITIS	4	26.91 (9.98 - 72.53)	4.72 (3.41)
	OESOPHAGOMEDIASTINAL FISTULA	3	345.12 (94.97 - 1254.1)	8.05 (6.42)
Nivolumab	PERITONITIS	63	2.54 (1.98 - 3.26)	1.34 (0.97)
	SMALL INTESTINAL PERFORATION	18	5.48 (3.43 - 8.74)	2.42 (1.75)
	LARGE INTESTINE PERFORATION	60	5.15 (3.99 - 6.65)	2.34 (1.96)
	GASTROINTESTINAL PERFORATION	38	5.44 (3.95 - 7.51)	2.42 (1.95)
	DIVERTICULAR PERFORATION	10	3.32 (1.78 - 6.2)	1.72 (0.84)
	ACQUIRED TRACHEO-OESOPHAGEAL FISTULA	12	88.74 (45.43 - 173.33)	5.99 (5.08)
	INTESTINAL PERFORATION	75	3.93 (3.13 - 4.94)	1.96 (1.62)
	APPENDICITIS PERFORATED	10	2.42 (1.3 - 4.51)	1.26 (0.39)
	DUODENAL PERFORATION	9	4.88 (2.52 - 9.45)	2.26 (1.34)
	ENTEROVESICAL FISTULA	7	6.21 (2.93 - 13.16)	2.6 (1.57)
	PNEUMOPERITONEUM	11	3.63 (2 - 6.58)	1.84 (1)
	JEJUNAL PERFORATION	5	11.55 (4.7 - 28.39)	3.46 (2.25)
	OESOPHAGEAL PERFORATION	14	8.9 (5.22 - 15.18)	3.1 (2.34)

	GASTRIC PERFORATION	19	3.83 (2.43 - 6.03)	1.92 (1.27)
	COLONIC FISTULA	4	4.09 (1.52 - 10.99)	2.01 (0.71)
	GASTROINTESTINAL ANASTOMOTIC LEAK	3	4.08 (1.3 - 12.79)	2.01 (0.55)
	OESOPHAGEAL FISTULA	4	7.85 (2.9 - 21.29)	2.93 (1.61)
	ILEAL PERFORATION	3	3.15 (1.01 - 9.86)	1.64 (0.19)
Pembrolizumab	PERITONITIS	56	2.34 (1.8 - 3.05)	1.22 (0.84)
	LARGE INTESTINE PERFORATION	59	5.26 (4.06 - 6.81)	2.37 (1.99)
	DUODENAL PERFORATION	19	10.97 (6.93 - 17.39)	3.39 (2.73)
	SMALL INTESTINAL PERFORATION	51	16.89 (12.71 - 22.45)	3.98 (3.57)
	OESOPHAGEAL FISTULA	3	6.06 (1.93 - 19.08)	2.57 (1.1)
	GASTROINTESTINAL PERFORATION	81	12.4 (9.92 - 15.51)	3.56 (3.24)
	INTESTINAL PERFORATION	95	5.2 (4.25 - 6.38)	2.35 (2.05)
	OESOPHAGEAL PERFORATION	12	7.88 (4.43 - 14.01)	2.94 (2.12)
	ACQUIRED TRACHEO-OESOPHAGEAL FISTULA	10	72.01 (35.4 - 146.48)	5.78 (4.81)
	JEJUNAL PERFORATION	6	14.55 (6.38 - 33.21)	3.78 (2.65)
	GASTRIC PERFORATION	17	3.55 (2.2 - 5.74)	1.81 (1.13)
	OESOPHAGOBRONCHIAL FISTULA	8	49.82 (23.2 - 106.99)	5.36 (4.31)
	INTESTINAL FISTULA	14	6.61 (3.89 - 11.25)	2.69 (1.93)
	GASTROINTESTINAL ANASTOMOTIC LEAK	3	4.24 (1.35 - 13.29)	2.06 (0.6)
	COLONIC FISTULA	3	3.17 (1.01 - 9.91)	1.65 (0.2)
	COLONIC ABSCESS	5	2.47 (1.02 - 5.96)	1.29 (0.11)
	GASTROINTESTINAL FISTULA	6	5.05 (2.25 - 11.33)	2.31 (1.21)
	RETROPERITONEAL ABSCESS	3	7.68 (2.43 - 24.27)	2.9 (1.43)
	ENTEROVESICAL FISTULA	9	8.36 (4.3 -	3.02 (2.09)

			16.26)	
Ipilimumab	ILEAL PERFORATION	3	3.28 (1.05 - 10.24)	1.7 (0.24)
	PNEUMOPERITONEUM	10	3.42 (1.83 - 6.38)	1.76 (0.88)
	RECTAL PERFORATION	12	14.71 (8.21 - 26.37)	3.8 (2.97)
	ABDOMINAL ABSCESS	5	3.27 (1.36 - 7.88)	1.71 (0.53)
	GASTROINTESTINAL PERFORATION	14	10.61 (6.27 - 17.96)	3.39 (2.65)
	LARGE INTESTINE PERFORATION	28	12.76 (8.79 - 18.52)	3.66 (3.12)
	INTESTINAL PERFORATION	19	5.29 (3.37 - 8.3)	2.4 (1.75)
	PERITONITIS	19	4.09 (2.61 - 6.42)	2.03 (1.38)
	SMALL INTESTINAL PERFORATION	5	8.03 (3.33 - 19.35)	3 (1.81)
	OESOPHAGOBRONCHIAL FISTULA	3	85.12 (26.38 - 274.62)	6.31 (4.81)
	DIVERTICULAR PERFORATION	3	5.3 (1.7 - 16.46)	2.4 (0.95)
	GASTRIC PERFORATION	5	5.35 (2.22 - 12.88)	2.41 (1.23)
	ACQUIRED TRACHEO-OESOPHAGEAL FISTULA	6	198.63 (83.69 - 471.45)	7.41 (6.24)

Table 2 Signal strength of immune checkpoint inhibitor–associated gastrointestinal ulceration adverse events in female.

Drug	Preferred term (PTs)	N	ROR（95%CI）	IC (IC025)
Atezolizumab	LARGE INTESTINE PERFORATION	9	6.93 (3.6 - 13.36)	2.78 (1.87)
	SMALL INTESTINAL PERFORATION	8	24.75 (12.26 - 49.97)	4.59 (3.62)
	GASTRIC PERFORATION	4	7.2 (2.69 - 19.25)	2.84 (1.54)
	ABDOMINAL ABSCESS	5	5.28 (2.19 - 12.71)	2.39 (1.21)
	INTESTINAL PERFORATION	11	4.74 (2.62 - 8.57)	2.24 (1.4)
	PERITONITIS	12	4.34 (2.46 - 7.66)	2.11 (1.31)
	GASTROINTESTINAL ANASTOMOTIC LEAK	4	63.39 (23 - 174.72)	5.89 (4.55)

	ENTEROVESICAL FISTULA	3	30.11 (9.53 - 95.12)	4.87 (3.39)
	GASTROINTESTINAL PERFORATION	5	6.39 (2.65 - 15.4)	2.67 (1.48)
Avelumab	NA	NA	NA	NA
Cemiplimab	NA	NA	NA	NA
Durvalumab	LARGE INTESTINE PERFORATION	4	7.72 (2.89 - 20.61)	2.94 (1.65)
	INTESTINAL PERFORATION	5	5.4 (2.25 - 13)	2.43 (1.25)
Ipilimumab	LARGE INTESTINE PERFORATION	8	15.6 (7.78 - 31.28)	3.95 (2.99)
	INTESTINAL PERFORATION	5	5.44 (2.26 - 13.09)	2.44 (1.26)
	PERITONITIS	5	4.57 (1.9 - 10.98)	2.19 (1.01)
	ACQUIRED TRACHEO-OESOPHAGEAL FISTULA	3	1715.66 (383.9 - 7667.33)	9.94 (8.17)
	SMALL INTESTINAL PERFORATION	3	23.11 (7.41 - 72.07)	4.52 (3.06)
Nivolumab	GASTROINTESTINAL PERFORATION	8	4.16 (2.07 - 8.34)	2.04 (1.07)
	ENTEROCUTANEOUS FISTULA	3	6.9 (2.2 - 21.62)	2.76 (1.3)
	INTESTINAL PERFORATION	22	3.86 (2.53 - 5.87)	1.94 (1.33)
	LARGE INTESTINE PERFORATION	20	6.3 (4.05 - 9.8)	2.63 (2)
	PERITONITIS	20	2.94 (1.89 - 4.56)	1.55 (0.91)
	DIVERTICULAR PERFORATION	6	7.12 (3.18 - 15.98)	2.81 (1.71)
	OESOPHAGEAL PERFORATION	3	9.07 (2.88 - 28.51)	3.15 (1.68)
	SMALL INTESTINAL PERFORATION	5	6.2 (2.56 - 15)	2.61 (1.42)
	GASTRIC PERFORATION	4	2.91 (1.09 - 7.79)	1.54 (0.24)
Pembrolizumab	PERITONITIS	32	3.22 (2.27 - 4.56)	1.67 (1.17)
	ABDOMINAL ABSCESS	8	2.34 (1.16 - 4.69)	1.22 (0.25)
	INTESTINAL PERFORATION	64	7.81 (6.09 - 10.01)	2.92 (2.56)
	GASTROINTESTINAL PERFORATION	49	18.42 (13.78 - 24.63)	4.11 (3.68)

LARGE INTESTINE PERFORATION	29	6.27 (4.33 - 9.06)	2.62 (2.08)
INTESTINAL FISTULA	11	12.46 (6.8 - 22.82)	3.57 (2.72)
SMALL INTESTINAL PERFORATION	30	27.69 (18.99 - 40.38)	4.64 (4.1)
DUODENAL PERFORATION	12	19.67 (10.93 - 35.4)	4.19 (3.36)
GASTROINTESTINAL FISTULA	6	11.24 (4.96 - 25.46)	3.43 (2.31)
GASTRIC PERFORATION	7	3.49 (1.66 - 7.37)	1.79 (0.76)
ENTEROVESICAL FISTULA	4	11.19 (4.11 - 30.48)	3.43 (2.1)
PNEUMOPERITONEUM	7	5.95 (2.81 - 12.59)	2.54 (1.51)
RECTAL PERFORATION	12	42.7 (23.15 - 78.79)	5.19 (4.33)

Table 3 Signal strength of immune checkpoint inhibitor–associated gastrointestinal ulceration adverse events in male.

Drug	Preferred term (PTs)	N	ROR（95%CI）	IC (IC025)
Atezolizumab	ILEAL PERFORATION	4	14.73 (5.42 - 40.01)	3.83 (2.51)
	DIVERTICULAR PERFORATION	5	6.67 (2.75 - 16.15)	2.72 (1.52)
	PERITONITIS	22	3.19 (2.1 - 4.86)	1.67 (1.06)
	LARGE INTESTINE PERFORATION	22	7.05 (4.63 - 10.76)	2.79 (2.19)
	GASTROINTESTINAL PERFORATION	16	10.16 (6.18 - 16.7)	3.31 (2.6)
	INTESTINAL PERFORATION	12	2.76 (1.56 - 4.86)	1.46 (0.65)
	SMALL INTESTINAL PERFORATION	4	4.06 (1.51 - 10.87)	2.01 (0.71)
	ANAL ABSCESS	10	2.65 (1.42 - 4.93)	1.4 (0.52)
	ANAL FISTULA	9	3.07 (1.59 - 5.91)	1.61 (0.69)
	APPENDICITIS PERFORATED	4	3.88 (1.45 - 10.38)	1.94 (0.64)
	DUODENAL PERFORATION	6	11.16 (4.95 - 25.14)	3.44 (2.33)
	RECTAL PERFORATION	3	12.7 (4.02 -	3.62 (2.15)

			40.13)	
	GASTRIC PERFORATION	7	5.36 (2.54 - 11.3)	2.4 (1.38)
	SPONTANEOUS BACTERIAL PERITONITIS	5	31.2 (12.52 - 77.75)	4.85 (3.62)
Avelumab	NA	NA	NA	NA
Cemiplimab	NA	NA	NA	NA
Durvalumab	LARGE INTESTINE PERFORATION	4	7.72 (2.89 - 20.61)	2.94 (1.65)
	INTESTINAL PERFORATION	5	5.4 (2.25 - 13)	2.43 (1.25)
Ipilimumab	ABDOMINAL ABSCESS	5	4.3 (1.78 - 10.35)	2.1 (0.91)
	LARGE INTESTINE PERFORATION	15	8.21 (4.93 - 13.67)	3.02 (2.29)
	INTESTINAL PERFORATION	11	4.34 (2.4 - 7.85)	2.11 (1.27)
	PERITONITIS	13	3.23 (1.87 - 5.57)	1.69 (0.91)
	GASTROINTESTINAL PERFORATION	10	10.8 (5.78 - 20.18)	3.41 (2.53)
	GASTRIC PERFORATION	4	5.23 (1.95 - 13.98)	2.38 (1.08)
	ACQUIRED TRACHEO-OESOPHAGEAL FISTULA	3	65.42 (19.93 - 214.78)	5.89 (4.36)
Nivolumab	PERITONITIS	40	1.91 (1.4 - 2.62)	0.93 (0.47)
	SMALL INTESTINAL PERFORATION	9	3.03 (1.56 - 5.87)	1.58 (0.65)
	LARGE INTESTINE PERFORATION	37	3.93 (2.84 - 5.46)	1.94 (1.47)
	ACQUIRED TRACHEO-OESOPHAGEAL FISTULA	11	63.15 (30.45 - 130.97)	5.38 (4.41)
	INTESTINAL PERFORATION	47	3.61 (2.7 - 4.83)	1.82 (1.4)
	DUODENAL PERFORATION	7	4.28 (2.02 - 9.1)	2.06 (1.02)
	GASTROINTESTINAL PERFORATION	22	4.62 (3.02 - 7.07)	2.17 (1.55)
	JEJUNAL PERFORATION	3	6.23 (1.95 - 19.9)	2.58 (1.09)
	OESOPHAGEAL PERFORATION	8	5.3 (2.61 - 10.76)	2.36 (1.37)
	GASTRIC PERFORATION	14	3.56 (2.09 - 6.06)	1.8 (1.05)
	PNEUMOPERITONEUM	10	3.72 (1.98 - 6.98)	1.86 (0.98)
	COLONIC FISTULA	3	4.2 (1.33 - 13.3)	2.03 (0.56)
	GASTROINTESTINAL	3	4.41 (1.39 -	2.1 (0.62)

	ANASTOMOTIC LEAK		13.96)	
	ENTEROVESICAL FISTULA	6	5.93 (2.61 - 13.46)	2.51 (1.39)
Pembrolizumab	LARGE INTESTINE PERFORATION	28	4.01 (2.75 - 5.83)	1.98 (1.43)
	DUODENAL PERFORATION	7	5.81 (2.73 - 12.35)	2.5 (1.46)
	SMALL INTESTINAL PERFORATION	21	9.93 (6.39 - 15.43)	3.23 (2.6)
	GASTROINTESTINAL PERFORATION	31	8.98 (6.25 - 12.89)	3.1 (2.57)
	PERITONITIS	24	1.55 (1.04 - 2.31)	0.63 (0.05)
	INTESTINAL PERFORATION	30	3.1 (2.16 - 4.44)	1.61 (1.09)
	OESOPHAGEAL PERFORATION	10	9.09 (4.81 - 17.18)	3.11 (2.22)
	ACQUIRED TRACHEO-OESOPHAGEAL FISTULA	9	64 (29.61 - 138.32)	5.53 (4.49)
	JEJUNAL PERFORATION	6	17.84 (7.68 - 41.44)	4.02 (2.87)
	GASTRIC PERFORATION	9	3.07 (1.59 - 5.94)	1.6 (0.68)
	OESOPHAGOBRONCHIAL FISTULA	5	30.29 (11.66 - 78.65)	4.68 (3.4)
	COLONIC ABSCESS	3	3.32 (1.06 - 10.4)	1.71 (0.25)
	FISTULA OF SMALL INTESTINE	3	4.91 (1.56 - 15.47)	2.26 (0.79)
	RETROPERITONEAL ABSCESS	3	18.87 (5.71 - 62.35)	4.09 (2.55)
	ENTEROVESICAL FISTULA	5	6.65 (2.72 - 16.26)	2.68 (1.48)

Table 4 Signal strength of immune checkpoint inhibitor–associated gastrointestinal ulceration adverse events in different genders.

DRUG	Preferred term (PTs)	N	ROR（95%CI）	IC (IC025)	P value	Adjusted p value
Atezolizumab	Large Intestine Perforation	9/22	0.61(0.28-1.33)	0.28	0.28	1
	Small Intestinal Perforation	8/4	2.98(0.9-9.91)	0.11	0.11	1
	Gastric Perforation	4/7	0.85(0.25-2.91)	1	1	1
	Intestinal Perforation	11/12	1.37(0.6-3.1)	0.59	0.59	1
	Peritonitis	12/22	0.81(0.4-1.64)	0.69	0.69	1
	Gastrointestinal Perforation	5/16	0.47(0.17-1.27)	0.19	0.19	1
Durvalumab	Large Intestine Perforation	4/5	1.56(0.42-5.82)	0.75	0.75	1

	Intestinal Perforation	5/9	1.08(0.36-3.24)	1	1	1
Ipilimumab	Large Intestine Perforation	8/15	1.17(0.5-2.77)	0.89	0.89	1
	Intestinal Perforation	5/11	1(0.35-2.88)	1	1	1
	Peritonitis	5/13	0.85(0.3-2.37)	0.95	0.95	1
	Acquired Tracheo-Oesophageal Fistula	3/3	2.2(0.44-10.91)	0.58	0.58	1
Nivolumab	Gastrointestinal Perforation	8/22	0.67(0.3-1.5)	0.43	0.43	1
	Intestinal Perforation	22/47	0.86(0.52-1.43)	0.65	0.65	1
	Large Intestine Perforation	20/37	0.99(0.58-1.71)	1	1	1
	Peritonitis	20/40	0.92(0.54-1.57)	0.86	0.86	1
	Oesophageal Perforation	3/8	0.69(0.18-2.6)	0.81	0.81	1
	Small Intestinal Perforation	5/9	1.02(0.34-3.05)	1	1	1
	Gastric Perforation	4/14	0.52(0.17-1.6)	0.36	0.36	1
Pembrolizumab	Peritonitis	32/24	1.23(0.73-2.1)	0.52	0.52	1
	Intestinal Perforation	64/30	1.98(1.28-3.05)	0	＜0.01	＜0.01
	Gastrointestinal Perforation	49/31	1.46(0.93-2.3)	0.12	0.12	1
	Large Intestine Perforation	29/28	0.96(0.57-1.61)	0.98	0.98	1
	Small Intestinal Perforation	30/21	1.32(0.76-2.31)	0.4	0.4	1
	Duodenal Perforation	12/7	1.59(0.62-4.03)	0.45	0.45	1
	Gastric Perforation	7/9	0.72(0.27-1.93)	0.69	0.69	1
	Enterovesical Fistula	4/5	0.74(0.2-2.76)	0.91	0.91	1

Table 5 Signal detection for ICI-associated gastrointestinal ulceration.

	N	ROR	ROR 95%CI	IC	IC025
Atezolizumab	243	3.01	2.65 - 3.41	1.58	1.40
Durvalumab	73	1.75	1.39 - 2.2	0.8	0.47
Pembrolizumab	540	2.35	2.15 - 2.55	1.22	1.10
Ipilimumab	305	4.43	3.95 - 4.95	2.13	1.97

Table 6 Signal Strength of Immune Checkpoint Inhibitor–Associated Gastrointestinal ulceration Events at the Preferred Term (PT) Level.

	Preferred term (PTs)	N	ROR（95%CI）	IC (IC025)
Atezolizumab	LARGE INTESTINE PERFORATION	30	5.82 (4.06 - 8.33)	2.53 (2.01)
	PERITONITIS	29	1.84 (1.28 - 2.65)	0.88 (0.35)
	GASTROINTESTINAL PERFORATION	28	9.08 (6.26 - 13.17)	3.17 (2.63)
	INTESTINAL PERFORATION	29	3.51 (2.44 - 5.05)	1.81 (1.28)
	FISTULA OF SMALL INTESTINE	3	11.02 (3.53 - 34.34)	3.45 (2)
	DIVERTICULAR PERFORATION	6	3.74 (1.68 - 8.33)	1.9 (0.81)
	OESOPHAGEAL PERFORATION	3	4.83 (1.55 - 15.01)	2.27 (0.82)
	SMALL INTESTINAL PERFORATION	10	7.34 (3.94 -	2.87 (1.99)

			13.66)	
	OESOPHAGEAL FISTULA	3	12.72 (4.08 - 39.69)	3.66 (2.2)
	GASTRIC ULCER PERFORATION	3	3.14 (1.01 - 9.76)	1.65 (0.2)
	GASTRIC PERFORATION	12	6.12 (3.47 - 10.78)	2.61 (1.8)
	DUODENAL PERFORATION	12	17.4 (9.84 - 30.76)	4.1 (3.29)
	GASTROINTESTINAL ANASTOMOTIC LEAK	5	16.97 (7.02 - 41.01)	4.07 (2.88)
	ANAL FISTULA	9	2.25 (1.17 - 4.32)	1.17 (0.25)
	ENTEROVESICAL FISTULA	3	5.39 (1.73 - 16.74)	2.42 (0.98)
	DIVERTICULITIS INTESTINAL PERFORATED	3	16.58 (5.3 - 51.8)	4.03 (2.58)
	SPONTANEOUS BACTERIAL PERITONITIS	6	42.77 (18.94 - 96.55)	5.37 (4.26)
Durvalumab	GASTROINTESTINAL PERFORATION	9	5.64 (2.93 - 10.85)	2.49 (1.58)
	OESOPHAGEAL PERFORATION	4	12.53 (4.69 - 33.46)	3.64 (2.34)
	INTESTINAL PERFORATION	13	3.05 (1.77 - 5.25)	1.61 (0.83)
	SPONTANEOUS BACTERIAL PERITONITIS	4	54.72 (20.3 - 147.48)	5.74 (4.43)
	OESOPHAGOMEDIASTINAL FISTULA	3	701.72 (193.11 - 2549.96)	9.08 (7.44)
Pembrolizumab	LARGE INTESTINE PERFORATION	59	4.03 (3.11 - 5.2)	2 (1.62)
	COLONIC ABSCESS	5	2.8 (1.16 - 6.75)	1.48 (0.3)
	DUODENAL PERFORATION	18	9.21 (5.77 - 14.69)	3.17 (2.51)
	ENTEROVESICAL FISTULA	9	5.71 (2.96 - 11.02)	2.5 (1.58)
	RECTAL PERFORATION	12	10.51 (5.92 - 18.63)	3.36 (2.55)
	GASTRIC PERFORATION	18	3.22 (2.03 - 5.12)	1.68 (1.02)
	OESOPHAGEAL PERFORATION	13	7.43 (4.3 - 12.86)	2.87 (2.09)
	INTESTINAL PERFORATION	99	4.22 (3.47 - 5.15)	2.07 (1.78)
	OESOPHAGOBRONCHIAL FISTULA	7	23.26 (10.87 - 49.78)	4.47 (3.42)
	SMALL INTESTINAL PERFORATION	46	12.09 (9.02 - 16.21)	3.56 (3.13)
	GASTROINTESTINAL FISTULA	7	3.75 (1.78 - 7.89)	1.9 (0.87)
	GASTROINTESTINAL PERFORATION	77	8.87 (7.08 - 11.11)	3.12 (2.79)

	ACQUIRED TRACHEO-OESOPHAGEAL FISTULA	10	69.18 (35.44 - 135.01)	5.9 (4.96)
	JEJUNAL PERFORATION	5	9.55 (3.93 - 23.16)	3.23 (2.03)
	INTESTINAL FISTULA	14	4.7 (2.78 - 7.96)	2.22 (1.47)
	GASTROINTESTINAL ANASTOMOTIC LEAK	3	3.55 (1.14 - 11.05)	1.82 (0.37)
	FISTULA OF SMALL INTESTINE	3	3.86 (1.24 - 12.03)	1.94 (0.49)
	RETROPERITONEAL ABSCESS	3	4.74 (1.52 - 14.79)	2.23 (0.78)
	PNEUMOPERITONEUM	9	2.35 (1.22 - 4.52)	1.23 (0.31)
Ipilimumab	INTESTINAL PERFORATION	10 8	15.42 (12.75 - 18.65)	3.93 (3.65)
	PERITONITIS	28	2.08 (1.43 - 3.01)	1.05 (0.52)
	GASTROINTESTINAL PERFORATION	30	11.38 (7.95 - 16.31)	3.5 (2.98)
	GASTRIC PERFORATION	7	4.16 (1.98 - 8.74)	2.05 (1.03)
	LARGE INTESTINE PERFORATION	77	17.61 (14.06 - 22.05)	4.12 (3.79)
	ABDOMINAL ABSCESS	8	2.87 (1.44 - 5.75)	1.52 (0.56)
	SMALL INTESTINAL PERFORATION	6	5.13 (2.3 - 11.44)	2.36 (1.26)
	JEJUNAL PERFORATION	3	18.94 (6.06 - 59.18)	4.23 (2.77)
	ACQUIRED TRACHEO-OESOPHAGEAL FISTULA	6	129.99 (56.32 - 299.99)	6.9 (5.75)

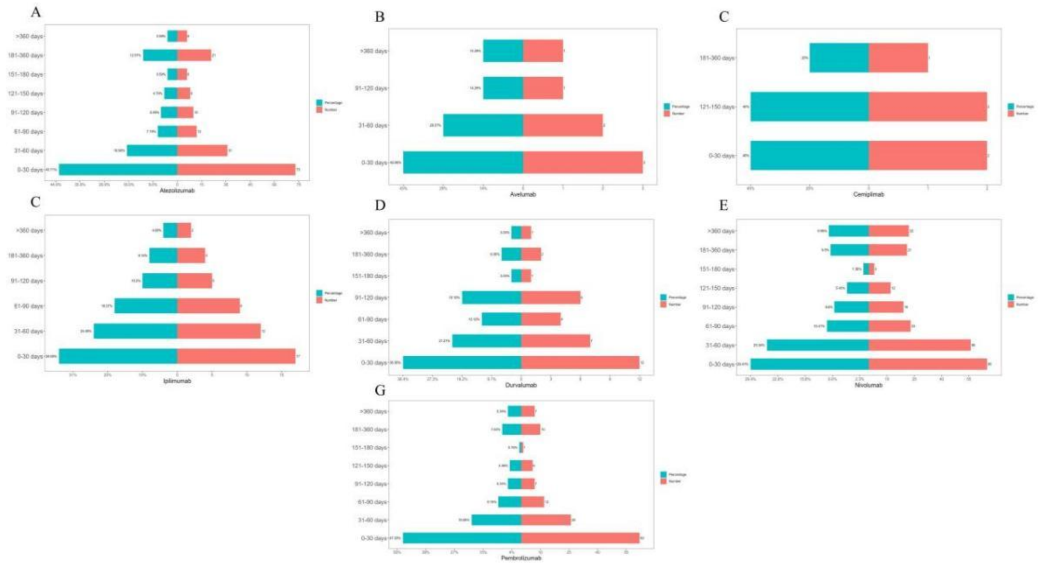

Figure 1 Time to onset of ICI-associated gastrointestinal ulceration AEs. (A) Atezolizumab; (B) Avelumab;(C)Cemiplimab;(D)Ipilimumab;(E)Durvalumab;(F)Nivolumab;(G)Pembrolizumab.
